# Multiscale modeling of composite cylindrical tank

**DOI:** 10.1016/j.dib.2018.04.091

**Published:** 2018-05-03

**Authors:** Eva Kormanikova, Kamila Kotrasova

**Affiliations:** Technical University of Kosice, Slovakia

## Abstract

This article provides the useful data information on multiscale modeling of liquid storage laminated composite cylindrical tank under seismic load. The investigation process is divided into three levels. Within the numerical homogenization, the hexagonal microstructure one-eighth and full RVE model is assumed on micro-scale level. Homogenization of unidirectional lamina is done in four steps. For effective material properties of the whole laminate, the laminated representative volume element is assumed on meso-scale level. The numerical homogenization is done by using the Finite Element Method in the program ANSYS. Effective modulus of elasticity is taken into the calculation of internal forces of the laminated composite cylindrical tank on macro-scale level. The base shears bottom of tank wall and tank base, the bending moment above the base plate, and overturning moment bellow the base plate as function of the tank fluid filling are presented. Data inform about seismic response of liquid laminated composite tank during seismic even in Slovak Republic, respecting of Eurocode 8 recommendations.

**Specifications table**

**Table:** 

Subject area	*Applied Mechanics*
More specific subject area	*Mechanics of Composite Materials, Fluid Dynamics*
Type of data	*Text file, graph, figure*
How data was acquired	*ANSYS software, Eurocode 8 - Design of structure for earthquake resistance - Part. 4: Silos, tanks and pipelines*
Data format	*Analyzed*
Experimental factors	*Numerical experiment*
Experimental features	*Numerical experiment of micro-mechanics of composite material and macro-mechanics of cylindrical tank*
Data source location	Slovakia, Europe
Data accessibility	*Data are available within this article*

**Value of the data**

• Approach of numerical homogenization of unidirectional lamina for the hexagonal microstructure.
• Interaction of fluid-laminated composite tank due to earthquake.
• Comparison of base shears of tank wall bottom and tank bottom as function of the tank fluid filling.
• Comparison of the bending moments above the base plate and overturning moments bellow the base plate depending on the tank fluid filling.

## 1 Data

### 1.1 Data on micro-scale level

On the micro-scale level, the material properties of one laminated composite layer are obtained for an unidirectional fiber reinforced composite layer consists of isotropic fibers and isotropic matrix. The fiber volume fraction and fiber diameter were found from electron microscope digital shot. Each layer of the laminate has the same thickness. The material properties of each layer were used from obtained average stresses [1], [2].

**Fig. 1:**
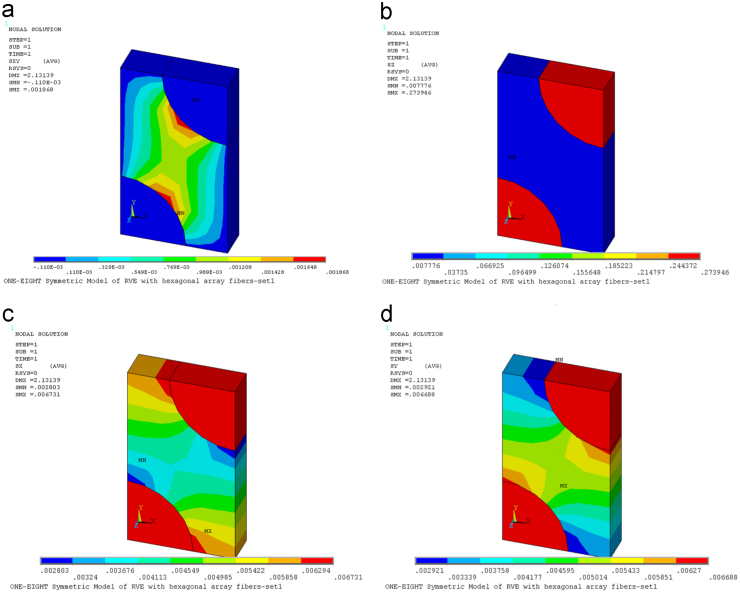
(a) Contour plot of stresses *τ*_*xy*_ on deformed RVE [TPa]. (b) Contour plot of stresses *σ*_*z*_ on deformed RVE [TPa]. (c) Contour plot of stresses *σ*_*x*_ on deformed RVE [TPa]. (d) Contour plot of stresses *σ*_*y*_ on deformed RVE [TPa].

**Fig. 2:**
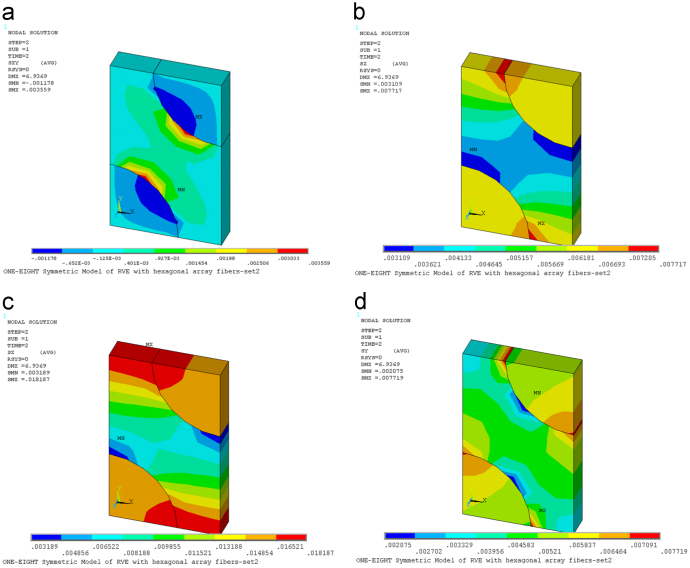
(a) Contour plot of stresses *τ*_*xy*_ on deformed RVE [TPa]. (b) Contour plot of stresses *σ*_*z*_ on deformed RVE [TPa]. (c) Contour plot of stresses *σ*_*x*_ on deformed RVE [TPa]. (d) Contour plot of stresses *σ*_*y*_ on deformed RVE [TPa].

**Fig. 3:**
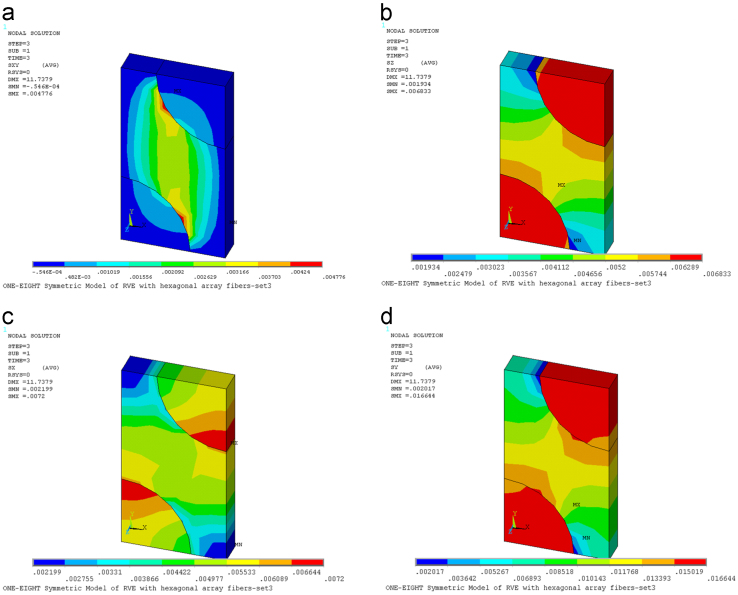
(a) Contour plot of stresses *τ*_*xy*_ on deformed RVE [TPa]. (b) Contour plot of stresses *σ*_*z*_ on deformed RVE [TPa]. (c) Contour plot of stresses *σ*_*x*_ on deformed RVE [TPa]. (d) Contour plot of stresses *σ*_*y*_ on deformed RVE [TPa].

**Fig. 4:**
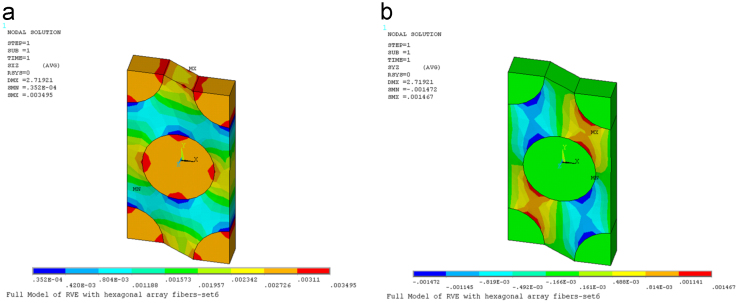
(a) Contour plot of stresses *τ*_xz_ on deformed RVE [TPa]. (b) Contour plot of stresses *τ*_*yz*_ on deformed RVE [TPa].

### 1.2 Data on meso-scale level

The circular cylindrical tank is made of the laminate [0/0/90/90]_*NS*_, where *N*=20 for the wall and for the base slab. The effective material properties of the whole laminate are observed from meso-scale level [1].

### 1.3 Data on macro-scale level

Then, these material properties are used for calculation of internal forces of laminated composite ground supported cylindrical tank [1], [3], [4]. The reservoir is filled with water. The container is without a roof slab. We consider only horizontal seismic load. A used elastic response spectrum is determined for the Slovak Republic, *a*_*g*_=1.5 m s^−2^, B category of the subsoil. The seismic response data of fluid filled laminated composite cylindrical tank was obtained.

**Fig. 5:**
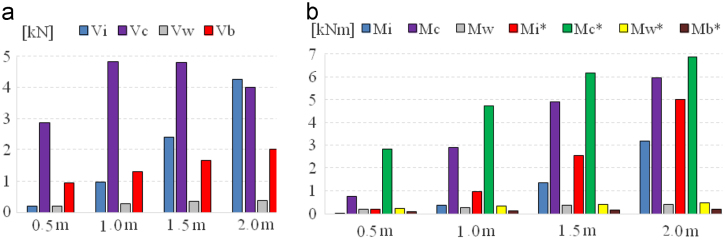
(a) Comparison of the base shears in impulsive and convective mode of fluid and the base shears of tank wall and tank bottom as function of the tank fluid filling, (b) comparison of the bending and overturning moments in impulsive and convective mode of fluid and tank wall and tank bottom depending on the tank fluid filling.

**Fig. 6:**
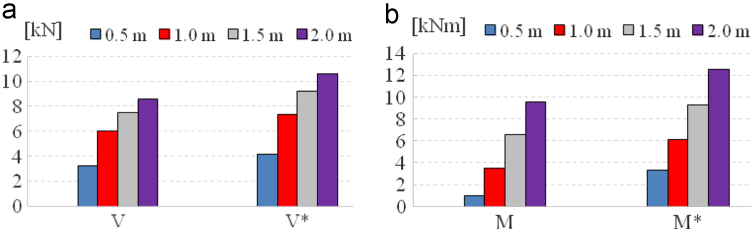
(a) Comparison of the total base shears of tank wall bottom and tank bottom as function of the tank fluid filling, (b) comparison of the total bending moments immediately above the base plate and total overturning moments.

## 2 Experimental design, materials and methods

For heterogeneous materials, a large number of material properties are needed [5]. The values of these properties change as a function of the volume fraction of reinforcement. An alternative to the experimental determination of these properties is an usage of homogenization techniques on representative volume element (RVE) [6], [7], [8], [9].

Within the numerical homogenization, the hexagonal microstructure was assumed. Four steps of numerical homogenization of unidirectional lamina were applied:

Step 1

In order to determine the components *C*_*i*1_ with *i*=1, 2, 3, (*z*, *x*, *y*), the strain $\varepsilon_{1}^{0}=1$ is applied to stretch the RVE in the fiber direction *x*_1_. The coefficients *C*_*i*1_ are found by using $C_{i1}={\bar{\sigma }}_{i}$.

Fig. 1 presents contour plot of stresses on deformed RVE under application of the strain $\varepsilon_{1}^{0}=1$.

Step 2

In order to determine the components *C*_*i*2_ with *i*=1, 2, 3, (*z*, *x*, *y*), the strain $\varepsilon_{2}^{0}=1$ is applied to stretch the RVE in the fiber direction *x*_2_. The coefficients *C*_*i*2_ are found by using $C_{i2}={\bar{\sigma }}_{i}$.

Fig. 2 presents contour plot of stresses on deformed RVE under application of the strain $\varepsilon_{2}^{0}=1$.

Step 3

In order to determine the components *C*_*i*3_ with *i*=1, 2, 3, (*z*, *x*, *y*), the strain $\varepsilon_{3}^{0}=1$ is applied to stretch the RVE in the fiber direction *x*_3_. The coefficients *C*_*i*3_ are found by using $C_{i3}={\bar{\sigma }}_{i}$.

Fig. 3 presents contour plot of stresses on deformed RVE under application of the strain $\varepsilon_{3}^{0}=1$.

Step 4

In order to determine the component C_66_, the strain $\gamma_{6}^{0}=\varepsilon_{12}^{0}+\varepsilon_{21}^{0}=1$ is applied to stretch the RVE. The coefficients *C*_55_ and *C*_66_ are found by using $C_{55}={\bar{\tau }}_{yz}$ and $C_{66}={\bar{\tau }}_{xz}$.

Fig. 4 presents contour plot of stresses on deformed RVE under application of the strain $\gamma_{6}^{0}=\varepsilon_{12}^{0}+\varepsilon_{21}^{0}=1$.

We considered different fluid filling heights of tank: 0.5 m, 1 m, 1.5 m and 2 m.

Fig. 5 presents:

– comparison of the base shears components in fluid impulsive mode and fluid convective mode, and the base shear components of the tank wall and tank bottom depending on the tank fluid filling,
– comparison of components of the bending and overturning moments in impulsive and convective mode of fluid, and moments components of tank wall and tank bottom as function of the tank fluid filling.

The total base shear at the bottom of the wall *V* consists of the sum *V*_*i*_, *V*_*c*_ and *V*_*w*_ and on the other side the total base shear at below the tank bottom V* comprises of the sum *V*_*i*_*, V*_*c*_*, V*_*w*_ and *V*_*b*_. The total bending moment at the bottom of the wall *M* consists of the sum *M*_*i*_*, M*_*c*_ and *M*_*w*_*,* and the total overturning moment immediately bellow of the tank base plate M* is given as the sum of *M*_*i*_*, M*_*c*_*, M*_*w*_ and *M*_*b*_. Index *“i”* means fluid impulsive mode component, *“c”* fluid convective mode component, *“w”* tank wall component, and “*b*” base bottom component.

Fig. 6 documents:

– comparison of the total base shears *V* of tank wall bottom and tank bottom V* as function of the tank fluid filling,
– comparison of the total bending moment immediately above the base plate *M* and total overturning moments immediately bellow the base plate M* depending on the tank fluid filling.
